# Emerging infectious diseases and biological invasions: a call for a One Health collaboration in science and management

**DOI:** 10.1098/rsos.181577

**Published:** 2019-03-13

**Authors:** Nick H. Ogden, John R. U. Wilson, David M. Richardson, Cang Hui, Sarah J. Davies, Sabrina Kumschick, Johannes J. Le Roux, John Measey, Wolf-Christian Saul, Juliet R. C. Pulliam

**Affiliations:** 1 National Microbiology Laboratory, Public Health Agency of Canada, Canada; 2 South African DST-NRF Centre of Excellence in Epidemiological Modelling and Analysis (SACEMA), Stellenbosch University, South Africa; 3 Centre for Invasion Biology, Department of Botany and Zoology, Stellenbosch University, South Africa; 4 South African National Biodiversity Institute, Kirstenbosch Research Centre, Claremont, Cape Town, South Africa; 5 Centre for Invasion Biology, Department of Mathematical Sciences, Stellenbosch University, Matieland 7602, South Africa; 6 Mathematical and Physical Biosciences, African Institute for Mathematical Sciences (AIMS), Muizenberg 7945, South Africa; 7 Department of Biological Sciences, Macquarie University, Sydney 2109, Australia

**Keywords:** biological invasion, emerging infectious disease, One Health

## Abstract

The study and management of emerging infectious diseases (EIDs) and of biological invasions both address the ecology of human-associated biological phenomena in a rapidly changing world. However, the two fields work mostly in parallel rather than in concert. This review explores how the general phenomenon of an organism rapidly increasing in range or abundance is caused, highlights the similarities and differences between research on EIDs and invasions, and discusses shared management insights and approaches. EIDs can arise by: (i) crossing geographical barriers due to human-mediated dispersal, (ii) crossing compatibility barriers due to evolution, and (iii) lifting of environmental barriers due to environmental change. All these processes can be implicated in biological invasions, but only the first defines them. Research on EIDs is embedded within the One Health concept—the notion that human, animal and ecosystem health are interrelated and that holistic approaches encompassing all three components are needed to respond to threats to human well-being. We argue that for sustainable development, biological invasions should be explicitly considered within One Health. Management goals for the fields are the same, and direct collaborations between invasion scientists, disease ecologists and epidemiologists on modelling, risk assessment, monitoring and management would be mutually beneficial.

## 1. Background

Changes to climate, habitats and biodiversity are affecting abiotic and biotic components of ecological niches, while social and economic changes (e.g. the development of megacities and increasing movement of people and goods in a globalized world) offer multiple routes for species translocation and dissemination [1–3]. Together these external drivers increasingly facilitate biological invasions, a major threat to biodiversity and ecosystems globally [4]. Non-native species include disease-causing microorganisms and parasites, and disease vectors (e.g. arthropod vectors such as mosquitoes), which pose substantial threats to human, domesticated animal and wildlife populations. Invasions by pathogens are, in public and animal health terms, emerging infectious diseases (EIDs; such as human immunodeficiency virus (HIV) and severe acute respiratory syndrome (SARS) [5,6]). In this paper, we focus on the mutual relevance of invasion science [7] and public health epidemiology in the context of EIDs of direct public health significance [8]. We also highlight how invasive non-pathogenic species, and infectious diseases that do not affect humans or domesticated animals directly, may indirectly impact human health. Possible indirect effects include those affecting the health of domesticated animals, crops, natural resources of wild plant and animal origin and also the health of natural ecosystems. Epidemiology is a broad field that encompasses many areas of health research; here, we use the term ‘epidemiologists’ to refer to those within the subspecialty focused on epidemiology of EIDs, which may also include disease ecologists. Responses to EIDs engage a wide community of medical, veterinary and public and animal health professionals.

The World Health Organization (WHO) defines an EID as ‘an infectious disease that has appeared in a population for the first time, or that may have existed previously but is rapidly increasing in incidence or geographic range’ (https://apps.who.int/iris/handle/10665/204722). Infectious diseases emerge via a number of mechanisms. ‘Adaptive emergence’ constitutes genetic change of a microorganism that results in a phenotype that is capable of invading a new ecosystem, particularly by jumping to new host species, including humans [9]. This mechanism of emergence may permit pathogens causing animal infections to become transmissible to humans (i.e. become zoonoses) and, in some cases, to be sustained by human-to-human transmission in the absence of animal reservoir hosts [10–12]. Expansion or ‘geographical emergence’ by changes to geographical ranges of pathogens or parasites can involve long-distance translocation, more localized spread or both. For invasion biologists, invasive species are those translocated intentionally or accidentally through a human agency (often over long distances) from the locations where they are native to an ecosystem where they were previously absent [13,14]. This is analogous to the emergence of EIDs by long-distance geographical spread.

The ideas that EIDs are essentially invasive species [15], and that two branches of science (invasion science and EID epidemiology) are studying similar phenomena [16–18], are not new. Furthermore, management objectives and methods may be similar [19]. Invasive arthropod vectors of parasites and pathogens, such as *Aedes* species of mosquitoes, are a case in point; they are traditionally considered part of EID studies, but are also studied by invasion biologists (e.g. [17,20]). However, despite these commonalities, functionally, the fields of invasion science and EID epidemiology work in parallel rather than together. Therefore, in this review, we explore the extent of similarities in key concepts, processes and methodological approaches, as well as useful differences that provide opportunities for synergies, which may enhance our understanding and practical management of invasions and EIDs. We call for these fields to be integrated within the One Health approach to enhance human well-being.

## 2. Common ground

### 2.1. Shared global context: the One Health concept

**Figure 1.**
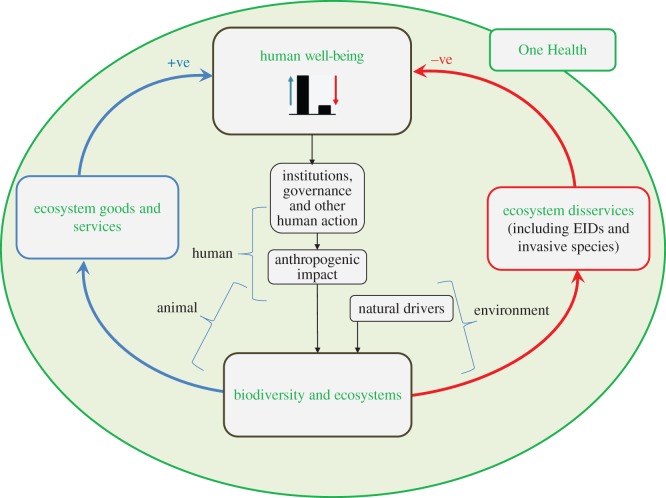
Biological invasions and EIDs as components of One Health. The schematic combines an adaptation of the IPBES Conceptual Framework [29] with a schematic of the One Health concept. The IPBES Conceptual Framework illustrates the interplay between anthropogenic and natural drivers of change in nature (biodiversity and ecosystems) (black boxes and arrows) and how this connects ecosystem services to human well-being (+ve effects, blue box and arrows). We also identify connections to ecosystem disservices, such as those caused by EIDs and invasive species (−ve effects, red box and arrows). For simplicity, positive effects of invasive species are not shown. The One Health concept (green circle) encompasses the IPBES Conceptual Framework, with its interacting human, animal and environment components.

EIDs that have affected humanity in recent decades have sharpened the focus of microbiologists, epidemiologists, human and animal health practitioners, as well as environmental and biological scientists, on the intersections of human, animal and ecosystem health. Emergence of many infectious diseases is associated with the dynamics of natural communities and their abiotic environmental determinants [21]. Many EIDs, including invasive pathogens such as West Nile virus (WNV) in North America, are maintained by (or originate in) wild animal hosts, and their emergence may have negative effects on natural communities as well as human or production animal health [22]. Accordingly, the One Health concept has evolved, which postulates that human, animal and ecosystem health are interrelated and interdependent, and that reactionary or preparatory responses to threats to human well-being demand holistic, transdisciplinary approaches encompassing all three components, including medical and veterinary practitioners and collaborators in ecosystem health [23]. Public health organizations around the world are increasingly adopting the One Health approach to make their responses to infectious diseases more effective (e.g. https://www.cdc.gov/onehealth/). The One Health concept encompasses benefits to human well-being (ecosystem services, i.e. benefits produced by ecosystem functions and structures for human well-being) as well as risks (ecosystem disservices, i.e. ‘nuisances’ for human well-being such as pests, and biological and geophysical hazards [24]). Both EIDs [25] and biological invasions [26] are important causes of ecosystem disservices, although biological invasions often render services and disservices at the same time [26]. Both disease emergence and biological invasions are increasing, being driven by the same global changes in climate, biodiversity, socio-economics and trade/travel [27]. The Intergovernmental Platform on Biodiversity and Ecosystem Services (IPBES, www.ipbes.net) was launched in 2012 to assess the state of biodiversity and of the ecosystem services it provides to society [28,29]. Integrating disservices in the IPBES conceptual framework illustrates the shared role that EIDs and biological invasions play for human well-being as components of One Health (figure 1).

**Figure 2.**
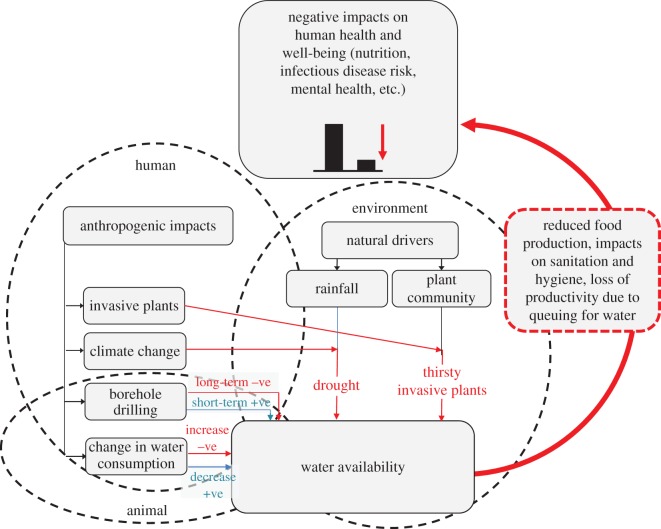
An example of indirect effects of invasive species on human health. Here, the indirect impact is water availability, which in South Africa is imperilled by invasive plants that are ‘thirsty’ (i.e. take up water at rates that significantly reduce water flows), climate change-induced drought and the competing requirements of drinking water for human populations, livestock production and other agricultural enterprises. How this issue is central to the One Health concept is illustrated by the interacting human, animal and environment components of the water availability problem as indicated by dashed circles. These circles indicate the main impacts of humans (the anthropogenic impacts), animals (the consumption of water by livestock and the consequent need to drill boreholes) and the environment (rainfall and plant communities).

The One Health concept recognizes that impacts of ecosystem changes on human health may act indirectly, e.g. via impacts on food and water security or by affecting biodiversity [17,30,31], and the UN Food and Agriculture Organization has adopted the One Health approach (http://www.fao.org/asiapacific/perspectives/one-health/en/). For example, several invasive trees in South Africa reduce water availability, thereby causing indirect impacts on human health (figure 2). More generally, biological invasions are increasingly being framed in a context of a transdisciplinary social–ecological system in which wider implications, including health and socio-economic impacts, are considered [32]. In South Africa, such transdisciplinary approaches have been termed ‘invasion science for society’ [33], which echoes the One Health concept.

### 2.2. Common drivers and biological processes

There are many overlaps and parallels between EIDs and biological invasions. Both involve species crossing geographical barriers that historically prevented natural dispersal, processes of establishment in a new environment, and subsequent range expansion to occupy the new environment. Not all EIDs can be termed invasive species, but some EIDs spread, and many establish, internationally, and such pathogens can be readily considered as invasive species (e.g. WNV, chikungunya, SARS and Zika in the Americas; chikungunya and dengue in Europe; HIV and influenzas globally). Even if EID emergence is associated with native range expansions (e.g. the spread of Lyme disease into Canada from the USA), and as such might not be formally considered as invasive species, insights on the basis of invasion concepts are still very relevant.

**Figure 3.**
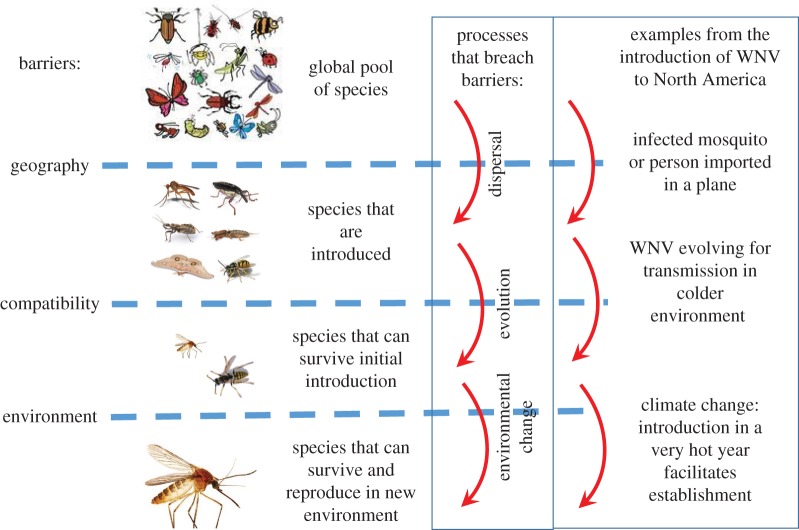
A conceptual diagram of the barriers to biological invasions and EIDs and how they limit species invasions and disease emergence. Processes whereby barriers may be breached are shown in the central box, and an example of these (from the introduction of West Nile virus (WNV) to North America) is shown in the box to the right. Note the only prerequisite for biological invasions is that there is dispersal across a geographical barrier (evolution and environmental change are not required if conditions are already suitable). By contrast, an EID can arise either through evolution leading to the breakdown in a compatibility barrier or environmental change breaking an environmental barrier without there being dispersal over a geographical barrier (cf. table 1). Moreover, the order of the barriers crossed can vary. For example, in the emergence of HIV, a compatibility barrier was first crossed (non-human primate to human) before the global spread of the pandemic. The insect collage used under ‘species that are introduced’ in Figure 3 was sourced from Wikimedia Commons under the Creative Commons Share-Alike License (CC-BY-SA 3.0; see https://commons.wikimedia.org/wiki/File:Insect_collage.png). We acknowledge the original author of the work: ‘BugBoy52.40’.

The concepts of ‘barriers’ and ‘stages’ are as relevant in biological invasions [13] as they are to disease emergence by international spread of a pathogen [5], and also to the processes mediating de novo emergence of a zoonosis from a microorganism maintained by animal reservoir hosts [34]. This topic has been reviewed before [17,35]. However, we focus on three key elements that permit, or prevent, EIDs and biological invasions: (i) geography, which is surmounted by dispersal; (ii) compatibility, which is determined by genetics and may be surmounted by evolution (including pre-adaptation via eco-evolutionary experience; see below); and (iii) environment, which is a barrier that may be lifted by disturbance, including environmental changes. Together, these factors mediate the biotic and abiotic qualities of the niche, the species' fitness in that niche and determine how the niche qualities and fitness may change (table 1 and figure 3).

(i) **Geography**: The crossing of historical geographical barriers and human-mediated introductions are related to both invasive species and many EIDs. The movement of invasive species, and long-distance dispersal of EIDs or their vectors, occurs via air and surface transport of goods and people [36]. For infectious diseases of humans, air travel is considered the most important route because it is rapid enough for humans infected in source locations to remain infective upon arrival in their destinations (e.g. SARS [37]). For many invasive species, the travel time from the native to the alien region is less important due to the occurrence of long-lived life stages such as seeds and eggs, so international spread of plants and animals is often facilitated by surface transport (on land or by sea). However, surface transport is also important for EIDs whereby infected arthropods, invasive arthropod vector eggs and infected animal hosts may be transported over long distances, e.g. the historical spread of plague and the recent spread of *Aedes albopictus* eggs/immatures in tyres and house plants [38–40]. While not a typical feature of EID introductions, deliberate transport and introduction of invasive species is common [41,42]. Also, both EIDs and invasive species have a history of, and the potential for, being introduced via the international pet trade [43,44], and both may be introduced deliberately as acts of bioterrorism (e.g. [45]). The bridging of the ‘geographical’ contact barrier between animals and humans (a process known as ‘spillover’) is essential for the de novo emergence of microorganisms as zoonoses, and the re-emergence of many zoonoses such as the spread of Nipah and Hendra viruses to humans (who are readily infected by the virus) from wildlife reservoirs [18]. Many zoonoses and arthropod vectors are dispersed regionally or more locally by natural means, which are not usually considered in the context of invasive species. Dispersal by migratory birds is one important mechanism whereby pathogens (e.g. influenza viruses) and some disease vectors (particularly ticks) can be dispersed over long distances (e.g. [46]).
  Beyond the simple contingency of species being transported into a new environment, the number and size of introduction events of a given species is also important. This is termed propagule pressure in invasion science and is analogous to concepts of infection frequency (relevant for spillover and introduction to new areas) and infective dose that are important in infectious disease epidemiology [13,17,47]. If propagule pressure is low, introduced species are more likely to undergo stochastic fade-out for a range of reasons, including the probability that an infected individual meets enough naive individuals for at least one of them to acquire infection (for infectious diseases), or to mate successfully (for any species undergoing sexual reproduction).
(ii) **Compatibility**: Both invasive species and EIDs must be capable of surviving in their new environment to the point of reproduction, and then of reproduction that supports stable or expanding populations. The capacity of an invading species to reproduce in the invaded environment is often measured as the intrinsic growth rate of the population (*r*, which is a time-based metric) in invasion science and the basic reproduction number (*R*_0_, which is a generation-based metric) in epidemiology. For persistence (i.e. naturalization) of invasive populations or EIDs, they must be compatible with ‘environmental’ conditions (including quantities such as host population size and density) to the extent that *r* is positive and *R*_0_ is greater than unity [15]. Whether or not an introduced organism becomes naturalized or invasive depends, to a great extent, on the eco-evolutionary experience of the introduced species and the recipient community. Eco-evolutionary experience describes the historical exposure of an organism to biotic interactions over evolutionary timescales [48,49], and emphasizes the role of traits selected for in previous environments (pre-adaptations), within both introduced and resident species, in driving the establishment success and adaptability of introduced species. In other words, eco-evolutionary experience determines the ease with which an invader can integrate into novel ecological contexts, and pre-adaptations are crucial determinants of a species' invasiveness and a community's invasibility [48–51]. Continuing evolutionary change of invading species is commonplace [52], and often involves admixture (intraspecific hybridization between previously allopatric populations) or hybridization between closely related species (e.g. [53]). Such genetic recombination often leads to enhanced performance by invasive populations due to heterosis and hybrid vigour [54]. However, many invasive species adapt in the absence of admixture or hybridization [54,55], resulting in traits that increase their performance. For example, invasive species may undergo rapid evolution in traits related to dispersal (e.g. [56]) and much insight has been gathered on such adaptations by identifying candidate genes underlying them. Adaptive emergence of EIDs for transmissibility of animal pathogens to or among humans explicitly requires genetic change, by mutations and recombination events [10,11]. However, as for non-disease-causing invasive species, pathogens and disease vectors continue to evolve and adapt to new environments into which they have been introduced, enhancing *R*_0_ within the invaded environments [57,58]. For pathogens of animals and humans, evolution towards increased *R*_0_ typically involves trade-offs between traits of transmission (higher pathogen loads mean more efficient transmission when contact is made between infected and naive hosts) and virulence (higher pathogen loads mean greater morbidity/mortality and reduced contact rates between infected and naive hosts) [59]. Such evolutionary processes are, however, highly idiosyncratic among pathogens that are transmitted by different routes [60] and among different populations [61]. Genetic changes may also permit invasive species and EIDs to persist long-term and not undergo ‘boom and bust’ which may occur for a range of reasons, including depletion of resources [62,63].
(iii) **Environment**: Environmental conditions determine whether a recipient location provides a suitable niche for species to establish and spread. Abiotic factors including climate (e.g. temperature, rainfall/humidity), and substrate qualities are key to whether introduced species can survive. Biotic factors, ranging from host population size, density and connectedness, and nutritional resources through ‘enemies’ (predators, parasites, pathogens, competitors and, for microorganisms, immunity and cross-immunity) to more complex community interactions, will determine whether introduced species can survive and reproduce [32,64]. When biogeographic barriers are breached by human action, species may be introduced to ecological niches that are suitable for their survival and reproduction and which also provide an ‘enemy-free’ space that further permits their establishment and spread. For this reason, the realized niche of species may be much larger in their introduced ranges than their native ranges [65]. The same is true of EIDs when they are introduced into an immunologically naive population [15]. While evolutionary change in invading species may alter the compatibility of the invading species with the invaded environment, environmental change may facilitate invasions by creating new suitable niches for invading species without the need for evolutionary change. Human disturbance of natural communities, ranging from replacement of natural vegetation with agricultural systems to more subtle changes, can make them more vulnerable to invasive species [66,67]. Such changes have similar effects on the process of emergence of infectious diseases in both wildlife and livestock [68]. Current and future global change (climate, biodiversity, landscape/land-use change, including urbanization) are likely to facilitate both disease emergence and biological invasions, while some sudden and unpredictable environmental fluctuations may inhibit invasions [69,70].

**Table 1.** Barriers to invasions and disease emergence, the processes whereby these may be surmounted and the phenomena and consequences that may result. EID, emerging infectious diseases.

initial barrier which when crossed can lead to the phenomenon	process	global change examples/mechanisms	EID examples
geographical	dispersal	biological invasions (i.e. inter-regional dispersal of alien species by humans)	EIDs involving international spread (e.g. HIV, SARS, WNV)
compatibility	evolution	pre-adaptation via eco-evolutionary experience. Evolution of new phenotypes in the environment (e.g. herbicide resistance, reduction in body size due to size-selected harvesting, new associations)	adaptive emergence of a zoonosis (e.g. zoonotic influenza) greater capacity to survive and reproduce, allowing species to spread (e.g. WNV in North America)
environmental	disturbance	land-use change that removes competitors or predators, or opens up resources allowing range expansion of species (native or non-native) climate change that changes the geographical location of the ecological niche of species	provides new opportunities for contact between humans, animals and disease vectors; and causes biodiversity change driving disease emergence diseases and their vectors (e.g. Lyme disease vectors in Canada)

In the above section, we have separated geographical, compatibility and environmental barriers, but they are often interdependent in influencing invasion/emergence (*r* and *R*_0_ depend on both compatibility and environment). Even when not mutually dependent, they act together. For example, environmental change (such as altered land use) can bridge the ‘geographical’ contact barrier between animal pathogens and humans, as is the case for Nipah virus [71]. Environmental changes also drive evolutionary changes that may alter the eco-evolutionary experience of potential invaders and potentially invaded communities. Issues of global spread of species and global environmental changes that drive disease emergence directly and indirectly (via non-disease-causing invasive species) underline the need for a One Health approach [23].

### 2.3. Similar methods

Risk analysis is a key management approach for both applied epidemiologists and invasion biologists. In this section, we focus primarily on risk assessment and return to discuss risk management later. Risk assessment is applied to help develop policies in anticipation of, and in response to, disease emergence events and biological invasions. To support these risk assessments, both disciplines aim to identify qualities (traits or syndromes) that (i) make species ‘invaders’ or ‘emergers’ (e.g. [72–74]), (ii) make source environments more likely to yield them (e.g. [74]), and (iii) render receiving environments susceptible or resistant to invaders or emerging pathogens [75]. Modelling is used in both invasion science and epidemiology to elucidate biological processes, predict establishment and spread, to support risk assessment and to assess effectiveness of interventions. The same ‘top-down’ (correlative, e.g. statistical models, ecological niche models and machine learning) and ‘bottom-up’ (mechanistic, e.g. dynamic simulation models, network analysis, individual-based models) methods are used for predicting the possible current and future extent of EIDs and invasive species [32,76]. Disease modelling methods used by epidemiologists would, of course, be directly relevant to modelling all types of infectious diseases, including those that affect species other than vertebrates, including plant pathogens [77]. Methods for monitoring invasive species, including active field surveillance and citizen science-based passive surveillance, have much in common with methods used to monitor risks from emerging zoonoses and vector-borne diseases in the environment [78–81]. Similar sampling designs are used and their implementation in target regions or sentinel sites is often determined by similar criteria, such as likely spread patterns predicted by species distribution and spread models, and occurrence of locations where impact may be greatest (e.g. [82]). In both disciplines, molecular approaches are used to confirm species identities and for source attribution [83,84], and both are exploring Earth observation data as proxies for potential occurrence of invaders [85], or risk from EIDs [86].

## 3. Useful differences: opportunities for synergies

### 3.1. Differences in scope

From an invasion biology perspective, EIDs are idiosyncratic in two ways. First, many important EIDs affecting humans and domesticated animals are obligate parasites of vertebrates [5], which means that consideration of the host population is paramount to predictive modelling and assessing impacts and risk. Parasitic species and microorganisms thus comprise a special subset of invasive species. For EIDs and parasitic invasive species, spread into naive populations may be rapid from the point of introduction to an epidemic, provided there is sufficient availability of naive hosts. To a first approximation, spread will not occur if the frequency of contact with naive hosts is below a threshold level. For microorganisms transmitted directly among humans, the patterns and extent of spread (equivalent to the ‘invasive range’) are mostly determined by characteristics of the human population and microorganism and not directly by the environment. The persistence of transmission cycles of microorganisms following spread (i.e. endemicity) depends on the details of the transmission characteristics of the microorganism and of the host population. As for non-infectious invasive species, emerging infections may boom and bust but usually due to mechanisms associated with the availability of susceptible hosts, through either reduction in the host population by a highly pathogenic EID or the development of immunity to the emerging pathogen in the host population [87].

Second, the causal organisms of EIDs (viruses, bacteria, fungi, protozoa and helminths) and vectors (particularly insects) are, for the most part, at the ‘small and fast’ end of the spectrum of invasive species, i.e. they have very small size and their generation time is often (but not always) short (days to months). By contrast, generation times may be years to decades for organisms like invasive trees. Notably, few invasive plants have reached their broad-scale climatic limits in their new ranges even centuries after introduction (e.g. [88–90]). Given the ease of accidental long-distance movement by human agency, microorganisms are likely to be common as invasive species of natural systems globally, although data on the occurrence of such events are very limited. Furthermore, due to their extremely short generation times, compared to many invasive plant species for example, they have greater capacity to adapt genetically to new environments. Despite this, and compared to their focus in EID epidemiology, microorganisms remain understudied in invasion biology due to a range of factors including difficulties with isolation or culturing, poorly known biogeography and therefore their native versus non-native status, and difficulties in detecting and ascribing impacts to the causative agent (e.g. [91]).

The first difference described above could be thought of as a limit on the scope of direct synergies in models used and the number of ‘invasive EIDs’ that may lend themselves to direct collaborations between invasion biologists and epidemiologists. However, clearly some invasive species are parasites or pathogens, and for these, the expertise of EID epidemiologists would enrich invasion biology. Furthermore, this apparent idiosyncrasy does not mean that invasion biologists cannot profit from modelling approaches developed in EID epidemiology. The second difference is of interest because the larger size (which makes their detection and enumeration easier) and longer generation times of many invasive species have meant that the demographic processes and community ecology of invasions have been more readily studied. Epidemiologists tend to use relatively simple criteria-led approaches or species distribution models to assess whether, and to what extent, invasion by pathogens and vectors may occur now and in the future (e.g. [92]). The approach to understanding the processes of introduction–naturalization–invasion used by invasion biologists has made it easier to describe and understand individual invasion processes [32]. This approach could be used to enhance risk assessment for EIDs, particularly those that are vector-borne and those that are zoonoses associated with wildlife, as all of the factors involved in these processes may determine the speed, trajectory and impact of EIDs as well as invasive species.

Factors that make species more successful invaders have been studied in invasion science since the 1980s (including using approaches of comparing native with invasive species, and invasive alien with alien-but-not-invasive species [93]), but only more recently by epidemiologists interested in emerging diseases [72,94,95]. Consequently, the elucidation of traits of invasiveness and invasibility and the recognition that these traits of invaders and invaded communities interact to permit or prevent invasions [96] is generally much richer than for EIDs. Studies in invasion science have led to concepts of traits that permit invaders to be more successful in certain environments (e.g. ‘urban winner’ species [97]), and ordination-type methods for classifying communities in terms of their invasibility (e.g. ‘periodic tables of niches' [98]). All of these could be a focus for direct knowledge transfer from invasion science to those assessing risk of zoonotic EIDs and arthropod vectors, and for conceptual exploration of their application to assessing risk of all EIDs. Ultimately, this may significantly enhance our understanding of the different components of the emergence/invasion systems allowing more effective prevention and control strategies.

### 3.2. Differences in risk management methods

As invasion biologists and epidemiologists have practical objectives of reducing impacts of the species that are their focus (by prevention, eradication, containment, control or impact reduction), sharing of tools, methods and activities that facilitate these objectives may have considerable value. This subject is worthy of a review in its own right—the following are simply examples.

While risk assessment of an anticipatory nature is very similar in the fields of infectious disease epidemiology and biological invasions, there are differences when risk management is conducted in the face of invasions or EIDs. In invasion science, risk management addresses the consequences of inaction by estimating the ‘invasion debt’, primarily of existing introduced species [99]. This approach could be readily adapted to risk management practices for EIDs. Those responsible for managing invasions use a range of tools, such as eradographs, to visualize the impacts of interventions to control geographical spread [100] and identification of management-specific switch points in control programmes that determine if and when management objectives should be changed [101].

Field surveillance/monitoring is conducted for both EIDs (particularly when these are zoonoses or vector-borne) and invasive species [102,103], and it may be practical and economical to develop combined field surveillance programmes. For example in Canada, south-to-north invasion of tick and fly vectors and of vector-borne pathogens of human and livestock health significance is occurring or a threat [102,103]. While the vectors and vector-borne diseases of livestock may not have human health importance, surveillance may use methods and/or locations similar enough for collaborations in field surveillance to be logical. Molecular methods are mainstream in identifying microbial pathogens in infectious disease surveillance programmes, but these methods are almost entirely used for identifying pathogens and comparisons to identify disease clusters or to attribute sources [104]. More detailed molecular analytical approaches are used in invasion science to understand invasion dynamics, such as underlying propagule pressure [105], landscape-scale dispersal patterns and rates [106], or to reconstruct invasion history and pathways [83,107]. These approaches may assist risk assessment and policies for management [108], while analysis of environmental DNA using meta DNA barcoding can assist in detecting any species (non-infectious invasive and EIDs) during transport, thereby aiding in preventing introductions from occurring [109]. While molecular approaches are often used to identify the provenance of source populations of invading populations and EIDs [84], they can also provide information relevant to biological control of invasive populations, for example, identifying the native regions where the prospects of identifying co-evolved biological control agents are more likely [110]. All of these more detailed approaches could be more widely implemented in the field of EID surveillance.

Passive citizen science methods of collecting information on species distributions are used both in public health and in ecology. In ecology, the object is monitoring of biogeography and global biodiversity information (e.g. eButterfly—http://www.e-butterfly.org/ and iSpot—https://www.ispotnature.org/). However, in public health, these methods have been developed to the point where data are systematically collected and analysed in national surveillance programmes to provide early warning of emerging vector-borne diseases allowing rapid responses [111]. Because most invasion science does not (directly) address human health issues, funding is probably much more difficult to mobilize for work on invasions than for EIDs. This means that cheaper means must be sought to detect new introduced species than can be implemented for EIDs. Nonetheless, the experience of public health epidemiologists in this area may benefit the field of invasion science, and epidemiologists may benefit from incorporating more cost-efficient methods developed in invasion biology.

In public health epidemiology, the need for rapid, specific and sensitive methods to detect clusters of disease cases as the first sign of an outbreak has led to a revolution in molecular and bioinformatics methods (particularly whole-genome sequencing and analysis) for species identification [104]. Given the potential for EID epidemics to arise rapidly, there has been considerable effort in public health to implement these molecular methods into programmes that systematically identify and control EIDs [111]. These complement data-driven international efforts to detect EID events including the joint WHO-OIE-FAO Global Early Warning System (GLEWS) for health threats and emerging risks at the human–animal–ecosystems interface (http://www.glews.net/), active detection of possible EID events via international media reports by the Global Public Health Intelligence Network (GPHIN), and passive detection of EID events by interested, voluntarily participating public health, infectious disease, veterinary, microbiology and academic experts in systems such as Promed (https://www.promedmail.org/) and Health Map (https://www.healthmap.org/en/) [111].

In general, control methods for EIDs (e.g. vaccines and quarantine) and invasive species (e.g. plant removal) are highly idiosyncratic, even if at first sight (such as chemical control of insects), they may seem very similar. However, despite these clear differences, prevention and control programmes for both EIDs and invasive species share the potential for interactions with the public to be crucial for programmes to succeed. Public trust and engagement (for example, in terms of personal and environmental impact, privacy/data-security, land ownership and access) may be essential for successful prevention and control [112]. Collaborations in developing procedures for public engagement may be very fruitful.

The often-rapid nature of disease emergence requires quick mobilization of expertise, and resources, including funding and personnel. The immediate relevance of EIDs to humans has united global efforts to counter them, and this has resulted in national and international networks of public health organizations coordinated (in the case of international outbreaks) by WHO. By contrast, calls for unity over invasions (e.g. [113]) have so far largely failed to produce effective agencies. There is, for example, no equivalent to public health organizations such as US Centers for Disease Control and Prevention, the Public Health Agency of Canada and the European Centre for Disease Prevention and Control that has prime responsibility for the detection and control of invasive species. This contrast is probably due to a number of reasons, including the local, regional or national (versus international) scope of many invasions, the often long time lag between biological invasions and detected impacts, and the generally slower nature of invasions, which together result in fractionated efforts that may be ineffective. Responsibilities for coordinating responses to EIDs of public health significance always lie with public health organizations, but responsibilities for responding to invasive species vary depending on the impact or location of the invasive species and may be organizations responsible for agriculture, fisheries, environment, natural resources, transport or local government entities [114].

## 4. Where to from here?

**Figure 4.**
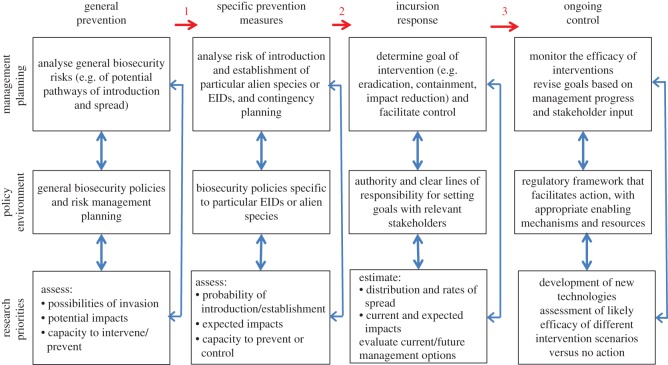
A One Health approach to the management of EIDs and biological invasions. The continuum of possible invasion/EID management functions, their policy or programme objectives, and the research activities that support their development are shown. The rows of boxes represent the different fields involved in responding to EIDs and biological invasions: management programmes, policy development and scientific research. The columns represent different stages of response to EIDs and invasions and how, as indicated by numbered red arrows, emphasis may change from general research into risks of EIDs and invasions, to focus on: (1) particular potential threats; (2) species/EIDs detected as invading; and (3) ongoing management of EIDs and invasive species.

Invasion science and epidemiology in the context of EIDs represent both applied and basic sciences. Both disciplines are involved in, and are informed by, fundamental research, and both have clear objectives and mandates to minimize negative impacts on society and the environment. The science is applied to the development of management plans along a continuum of points of potential anticipatory and responsive actions (figure 4). These functions are, to a greater or lesser extent, already undertaken independently by those involved in the study, prevention and control of EIDs and biological invasions. However, we advocate for a strong, collaborative One Health approach in these actions that integrates across human, animal and environmental health, including both invasion biology and epidemiology in the field of EIDs.

There are clear opportunities for immediate collaboration:

(1) Predictive modelling: Modelling of dispersal, introduction and spread of EIDs and invasive species would be a relatively simple point of collaboration as the objectives are similar. This would be applicable to all parts of the continuum of management functions, but particularly to anticipating the risk of invasions and EIDs.
(2) Monitoring of EIDs and invasions: International scanning, as conducted for EIDs, could be readily applied to biological invasions as has been proposed previously [115]. The increasing availability of ‘big data’ to support detection and monitoring of EIDs and biological invasions, and the challenges of analysing these data to provide intelligence, is a particularly needed avenue of collaborative action and research [115,116]. Collaborative monitoring (and more systematic surveillance) for EIDs and invasive species at points of entry, monitoring in field studies (including assessing indirect effects of invasions on health), and collaboration on the development and application of molecular methods for detection and demographic analysis of populations of invasive species and EIDs are all areas where synergistic activities could increase efficiency.
(3) Management of invasions and EIDs: Given the transferable skill sets between those involved in EID and invasive species management, and the possibilities for synergies between the fields, collaboration across the range of management activities could be very advantageous, recognizing that the responsibilities for managing biological invasions and EIDs may be distinct.

We are not aware of examples where the application of epidemiology to biological invasions or invasion biology to EIDs has resulted in improved outcomes in terms of prevention or control. However, the application of epidemiological modelling may well have contributed to understanding patterns of spread of chytridiomycosis in amphibians, which is very likely a transmissible disease (e.g. [117]). Similarly, while the emerald ash borer beetle and WNV (both invasive species) arrived almost simultaneously at the US–Canada border, to date practical spread modelling of the emerald ash borer as conducted by invasion biologists [118] has only been matched by theoretical mathematical modelling of the spread of WNV [119]. Furthermore, there is no cross-talk between those responsible for predicting spread and responding to the emerald ash borer (https://www.nrcan.gc.ca/forests/fire-insects-disturbances/top-insects/13377), and those responsible for responding to WNV (https://www.canada.ca/en/public-health/services/diseases/west-nile-virus/surveillance-west-nile-virus.html), when it is recognized that changes in biodiversity (such as those occurring as a consequence of the invasion of emerald ash borer) may have impacts on risk from WNV [120]. The lack of integration of these responses provides a clear example of a missed opportunity to benefit from the One Health framework.

Throughout, collaborations need to be win–win for epidemiologists and invasion biologists, and they need to be enabled. ‘Soft’ collaborations within the academic context would be the easiest to set up, and may only require simple encouragement (e.g. joint seminars, learning exchanges or workshops). More solid collaborations on joint projects, such as proposed for ‘Global networks for invasion science’ [121], would require collaborative funding opportunities. Possibly the most enabling step would be the development of common collaborative programmes founded on common policy initiatives of national and international organizations responsible for managing EIDs and biological invasions. In the One Health field, this has begun with the animal health, human health and food security organizations working collaboratively on the FAO/OIE/WHO Tripartite Collaboration on antimicrobial resistance (AMR: http://www.who.int/foodsafety/areas_work/antimicrobial-resistance/tripartite/en/). While human health has a UN organization, the WHO, that provides international leadership and coordination on EIDs, there is currently no analogous body for invasion science. The United Nations Environment Programme (UNEP) and IUCN's Invasive Species Specialist Group (ISSG) may be two of the most promising institutional bodies that could facilitate interactions between invasion biologists and epidemiologists (and their organizations).

## 5. Conclusion

The fields of invasion science and EID epidemiology share the challenge of the increasing numbers of invasions and EIDs with no evidence of saturation. Invasions and EIDs involve similar biological processes, may be intrinsically linked biologically and by human activity, are addressed by scientists with similar skills and objectives, and are being driven by the same global changes. Invasions by non-pathogenic organisms can also have important impacts on human health. They are therefore both part of the One Health concept and require a One Health approach to minimize their negative impacts on humanity. We have identified exciting opportunities for synergies between the fields of invasion science and EID epidemiology and call for greater collaboration to benefit humanity.

## Supplementary Material

Reviewer comments

## Data accessibility

There are no data associated with this review article.

## References

[1] Tatem AJ, Hay SI, Rogers DJ 2006 Global traffic and disease vector dispersal. Proc. Natl Acad. Sci. USA 103, 6242–6247. (10.1073/pnas.0508391103) 16606847 PMC1435368

[2] Hulme PE et al. 2008 Grasping at the routes of biological invasions: a framework for integrating pathways into policy. J. Appl. Ecol. 45, 403–414. (10.1111/j.1365-2664.2007.01442.x)

[3] Wilson JRU, Dormontt EE, Prentis PJ, Lowe AJ, Richardson DM 2009 Something in the way you move: dispersal pathways affect invasion success. Trends Ecol. Evol. 24, 136–144, (10.1016/j.tree.2008.10.007) 19178981

[4] Pyšek P, Richardson DM 2010 Invasive species, environmental change and management, and health. Annu. Rev. Environ. Resour. 35, 25–55. (10.1146/annurev-environ-033009-095548)

[5] Jones KE, Patel NG, Levy MA, Storeygard A, Balk D, Gittleman JL, Daszak P 2008 Global trends in emerging infectious diseases. Nature 451, 990–993. (10.1038/nature06536) 18288193 PMC5960580

[6] Pike J, Bogich T, Elwood S, Finnoff DC, Daszak P 2014 Economic optimization of a global strategy to address the pandemic threat. Proc. Natl Acad. Sci. USA 111, 18 519–18 523. (10.1073/pnas.1412661112) PMC4284561 25512538

[7] Richardson DM 2011 Invasion science: the roads travelled and the roads ahead. In Fifty years of invasion ecology: the legacy of Charles Elton (ed. Richardson DM), pp. 397–407. Oxford, UK: Wiley-Blackwell.

[8] Mackenbach JP 1995 Public health epidemiology. J. Epidemiol. Community Health 49, 333–334. (10.1136/jech.49.4.333) 7650454 PMC1060118

[9] Pepin KM, Lass S, Pulliam JR, Read AF, Lloyd-Smith JO 2010 Identifying genetic markers of adaptation for surveillance of viral host jumps. Nat. Rev. Microbiol. 8, 802–813. (10.1038/nrmicro2440) 20938453 PMC7097030

[10] Antia R, Regoes RR, Koella JC, Bergstrom CT 2003 The role of evolution in the emergence of infectious diseases. Nature 426, 658–661. (10.1038/nature02104) 14668863 PMC7095141

[11] Smith GJ et al. 2009 Origins and evolutionary genomics of the 2009 swine-origin H1N1 influenza A epidemic. Nature 459, 1122–1125. (10.1038/nature08182) 19516283

[12] Joseph U, Su YC, Vijaykrishna D, Smith GJ 2017 The ecology and adaptive evolution of influenza A interspecies transmission. Influenza Other Respir. Viruses 11, 74–84. (10.1111/irv.12412) 27426214 PMC5155642

[13] Blackburn TM, Pyšek P, Bacher S, Carlton JT, Duncan RP, Jarošík V, Wilson JR, Richardson DM 2011 A proposed unified framework for biological invasions. Trends Ecol. Evol. 26, 333–339. (10.1016/j.tree.2011.03.023) 21601306

[14] Wilson JRU, García-Díaz P, Cassey P, Richardson DM, Pyšek P, Blackburn TM 2016 Biological invasions and natural colonisations are different—the need for invasion science. NeoBiota 31, 87–98. (10.3897/neobiota.31.9185)

[15] Anderson RM, May RM 1986 The invasion, persistence and spread of infectious diseases within animal and plant communities. Phil. Trans. R. Soc. B 314, 533–570. (10.1098/rstb.1986.0072) 2880354

[16] Drake JM, Lodge DM 2006 Allee effects, propagule pressure and the probability of establishment: risk analysis for biological invasions. Biol. Invasions 8, 365–375. (10.1007/s10530-004-8122-6)

[17] Hatcher MJ, Dick JTA, Dunn AM 2012 Disease emergence and invasions. Funct. Ecol. 26, 1275–1287. (10.1111/j.1365-2435.2012.02031.x) PMC7163950 32313353

[18] Dunn AM, Hatcher MJ 2015 Parasites and biological invasions: parallels, interactions, and control. Trends Parasitol. 31, 189–199. (10.1016/j.pt.2014.12.003) 25613560

[19] Crowl TA, Crist TO, Parmenter RR, Belovsky G, Lugo AE 2008 The spread of invasive species and infectious disease as drivers of ecosystem change. Front. Ecol. Environ. 6, 238–246. (10.1890/070151)

[20] Dunn AM, Perkins SE 2012 Invasions and infections. Funct. Ecol. 26, 1365–2435. (10.1111/1365-2435.12022)

[21] Daszak P, Cunningham AA, Hyatt AD 2001 Anthropogenic environmental change and the emergence of infectious diseases in wildlife. Acta Trop. 78, 103–116. (10.1016/S0001-706X(00)00179-0) 11230820

[22] LaDeau SL, Kilpatrick AM, Marra PP 2007 West Nile virus emergence and large-scale declines of North American bird populations. Nature 447, 710–713. (10.1038/nature05829) 17507930

[23] Cunningham AA, Scoones I, Wood JLN 2017 Introduction: One Health for a changing world: new perspectives from Africa. Phil. Trans. R. Soc. B 372, 20160162 (10.1098/rstb.2016.0162) 28584170 PMC5468687

[24] von Döhren P, Haase D. 2015 Ecosystem disservices research: a review of the state of the art with a focus on cities. Ecol. Indic. 52, 490–497. (10.1016/j.ecolind.2014.12.027)

[25] Dunn RR 2010 Global mapping of ecosystems disservices: the unspoken reality that nature sometimes kills us. Biotropica 42, 555–557. (10.1111/j.1744-7429.2010.00698.x)

[26] Vaz AS et al. 2017 Integrating ecosystem disservices and services: insights from plant invasions. Ecosyst. Serv. 23, 94–107. (10.1016/j.ecoser.2016.11.017)

[27] Millennium Ecosystem Assessment. 2005 Millennium ecosystem assessment. Ecosystems and human wellbeing: a framework for assessment. Washington, DC: Island Press.

[28] Perrings C, Duraiappah A, Larigauderie A, Mooney H 2011 The biodiversity and ecosystem services science-policy interface. Science 331, 1139–1140. (10.1126/science.1202400) 21330488

[29] Diaz S et al. 2015 The IPBES conceptual framework—connecting nature and people. Curr. Opin. Environ. Sust. 14, 1–16. (10.1016/j.cosust.2014.11.002)

[30] Nabarro D 2012 One Health: towards safeguarding the health, food security and economic welfare of communities. Onderstep. J. Vet. Res. 79, 450 (10.4102/ojvr.v79i2.450) 23327369

[31] Murtaugh MP, Steer CJ, Sreevatsan S, Patterson E, Kennedy S, Sriramarao P 2017 The science behind One Health: at the interface of humans, animals, and the environment. Ann. NY Acad. Sci. 1395, 12–32. (10.1111/nyas.13355) 28505393

[32] Hui C, Richardson DM 2017 Invasion dynamics. Oxford, UK: Oxford University Press.

[33] Van Wilgen BW, Davies SJ, Richardson DM. 2014 Invasion science for society: a decade of contributions from the Centre for Invasion Biology. S. Afr. J. Sci. 110, a0074 (10.1590/sajs.2014/a0074)

[34] Lloyd-Smith JO, George D, Pepin KM, Pitzer VE, Pulliam JRC, Dobson AP, Hudson PJ, Grenfell BT 2009 Epidemic dynamics at the human–animal interface. Science 326, 1362–1367. (10.1126/science.1177345) 19965751 PMC3891603

[35] Jeschke JM, Keesing F, Ostfeld RS 2013 Novel organisms: comparing invasive species, GMOs, and emerging pathogens. Ambio 42, 541–548. (10.1007/s13280-013-0387-5) 23456779 PMC3698323

[36] Trakhtenbrot A, Nathan R, Perry G, Richardson DM 2005 The importance of long-distance dispersal in biodiversity conservation. Divers. Distrib. 11, 173–181. (10.1111/j.1366-9516.2005.00156.x)

[37] Naylor CD 2003 Learning from SARS: renewal of public health in Canada: a report of the national advisory committee on SARS and public health. Ottawa, Canada: National Advisory Committee on SARS and Public Health.

[38] Burgess NR 1995 *Aedes albopictus*: a potential problem in the United Kingdom. Parassitologia 37, 121–122. 8778653

[39] Medlock JM, Hansford KM, Schaffner F, Versteirt V, Hendrickx G, Zeller H, Van Bortel W. 2012 A review of the invasive mosquitoes in Europe: ecology, public health risks, and control options. Vector Borne Zoonotic Dis. 12, 435–447. (10.1089/vbz.2011.0814) 22448724 PMC3366101

[40] Dean KR, Krauer F, Walløe L, Lingjærde OC, Bramanti B, Stenseth NC, Schmid BV 2018 Human ectoparasites and the spread of plague in Europe during the Second Pandemic. Proc. Natl Acad. Sci. USA 115, 1304–1309. (10.1073/pnas.1715640115) 29339508 PMC5819418

[41] Hulme PE 2009 Trade, transport and trouble: managing invasive species pathways in an era of globalization. J. Appl. Ecol. 46, 10–18. (10.1111/j.1365-2664.2008.01600.x)

[42] Essl F et al. 2015 Crossing frontiers in tackling pathways of biological invasions. BioScience 65, 769–782. (10.1093/biosci/biv082)

[43] Marano N, Arguin PM, Pappaioanou M 2007 Impact of globalization and animal trade on infectious disease ecology. Emerg. Infect. Dis. 12, 1807–1809. (10.3201/eid1211.061013) PMC2876780 18258027

[44] Jenkins PT 2011 Pet trade. In Encyclopedia of biological invasions (eds Simberloff D, Rejmánek M), pp. 539–543. Berkeley, CA: University of California Press.

[45] Lockwood JA 2012 Insects as weapons of war, terror, and torture. Annu. Rev. Entomol. 57, 205–227. (10.1146/annurev-ento-120710-100618) 21910635

[46] Ogden NH et al. 2008 The role of migratory birds in introduction and range expansion of *Ixodes scapularis* ticks, and *Borrelia burgdorferi* and *Anaplasma phagocytophilum* in Canada. Appl. Environ. Microbiol. 74, 1780–1790. (10.1128/AEM.01982-07) 18245258 PMC2268299

[47] Lockwood JL, Cassey P, Blackburn T 2005 The role of propagule pressure in explaining species invasions. Trends Ecol. Evol. 20, 223–228. (10.1016/j.tree.2005.02.004) 16701373

[48] Saul W-C, Jeschke JM, Heger T 2013 The role of eco-evolutionary experience in invasion success. NeoBiota 17, 57–74. (10.3897/neobiota.17.5208)

[49] Saul W-C, Jeschke JM 2015 Eco-evolutionary experience in novel species interactions. Ecol. Lett. 18, 236–245. (10.1111/ele.12408) 25626585

[50] Maynard Smith J 1982 Evolution and the theory of games. Cambridge, UK: Cambridge University Press.

[51] Fridley JD, Sax DF 2014 The imbalance of nature: revisiting a Darwinian framework for invasion biology. Glob. Ecol. Biogeogr. 23, 1157–1166. (10.1111/geb.12221)

[52] Cox GW 2004 Alien species and evolution: the evolutionary ecology of exotic plants, animals, microbes, and interacting native species. Washington, DC: Island Press.

[53] Diedericks G, Henriques R, von der Heyden S, Weyl OLF, Hui C 2018 Sleeping with the enemy: introgressive hybridisation in two invasive centrarchids. J. Fish Biol. 93, 405–410. (10.1111/jfb.13730) 29959774

[54] Huey RB, Gilchrist GW, Carlson ML, Berrigan D, Serra L 2000 Rapid evolution of a geographic cline in size in an introduced fly. Science 287, 308–309. (10.1126/science.287.5451.308) 10634786

[55] Kolbe JJ, Glor RE, Schettino LR, Lara AC, Larson A, Losos JB 2004 Genetic variation increases during biological invasion by a Cuban lizard. Nature 31, 177–181. (10.1038/nature02807) 15356629

[56] Colautti RI, Barrett SCH 2013 Rapid adaptation to climate facilitates range expansion of an invasive plant. Science 342, 364–366. (10.1126/science.1242121) 24136968

[57] Moudy RM, Meola MA, Morin LL, Ebel GD, Kramer LD 2007 A newly emergent genotype of West Nile virus is transmitted earlier and more efficiently by *Culex* mosquitoes. Am. J. Trop. Med. Hyg. 77, 365–370. (10.4269/ajtmh.2007.77.365) 17690414

[58] Goubert C, Henri H, Minard G, Valiente Moro C, Mavingui P, Vieira C, Boulesteix M 2017 High-throughput sequencing of transposable element insertions suggests adaptive evolution of the invasive Asian tiger mosquito towards temperate environments. Mol. Ecol. 26, 3968–3981. (10.1111/mec.14184) 28517033

[59] Blanquart F et al. 2016 A transmission-virulence evolutionary trade-off explains attenuation of HIV-1 in Uganda. Elife 5, e20492 (10.7554/eLife.20492) 27815945 PMC5115872

[60] Leggett HC, Cornwallis CK, Buckling A, West SA 2017 Growth rate, transmission mode and virulence in human pathogens. Phil. Trans. R. Soc. B 372, 20160094 (10.1098/rstb.2016.0094) 28289261 PMC5352820

[61] Boots M, Hudson PJ, Sasaki A 2004 Large shifts in pathogen virulence relate to host population structure. Science 303, 842–844 (10.1126/science.1088542) 14764881

[62] Simberloff D, Gibbons L 2004 Now you see them, now you don't—population crashes of established introduced species. Biol. Invasions 6, 161–172. (10.1023/B:BINV.0000022133.49752.46)

[63] Strayer DL et al. 2017 Boom-bust dynamics in biological invasions: towards an improved application of the concept. Ecol. Lett. 20, 1337–1350. (10.1111/ele.12822) 28834087

[64] Wasserman S, Tambyah PA, Lim PL 2016 Yellow fever cases in Asia: primed for an epidemic. Int. J. Infect. Dis. 48, 98–103. (10.1016/j.ijid.2016.04.025) 27156836

[65] Elith J, Kearney M, Phillips S 2010 The art of modelling range-shifting species. Meth. Ecol. Evol. 1, 330–342. (10.1111/j.2041-210X.2010.00036.x)

[66] Hobbs RJ, Huenneke LF 1992 Disturbance, diversity, and invasion: implications for conservation. Conserv. Biol. 6, 324–337. (10.1046/j.1523-1739.1992.06030324.x)

[67] Woodford DJ, Hui C, Richardson DM, Weyl OLF 2013 Propagule pressure drives establishment of introduced freshwater fish: quantitative evidence from an irrigation network. Ecol. Appl. 23, 1926–1937 (10.1890/12-1262.1) 24555318

[68] Guo F, Bonebrake TC, Gibson L In press. Land-use change alters host and vector communities and may elevate disease risk. Ecohealth. (10.1007/s10393-018-1336-3) 29691680

[69] Altizer S, Ostfeld RS, Johnson PT, Kutz S, Harvell CD 2013 Climate change and infectious diseases: from evidence to a predictive framework. Science 341, 514–519. (10.1126/science.1239401) 23908230

[70] Faust CL, McCallum HI, Bloomfield LSP, Gottdenker NL, Gillespie TR, Torney CJ, Dobson AP, Plowright RK 2018 Pathogen spillover during land conversion. Ecol. Lett. 21, 471–483. (10.1111/ele.12904) 29466832

[71] Pulliam JR et al. 2012 Agricultural intensification, priming for persistence and the emergence of Nipah virus: a lethal bat-borne zoonosis. J. R. Soc. Interface 9, 89–101. (10.1098/rsif.2011.0223) 21632614 PMC3223631

[72] Pulliam JRC 2008 Viral host jumps: moving toward a predictive framework. EcoHealth 5, 80–91. (10.1007/s10393-007-0149-6) 18648800 PMC7087992

[73] Rejmánek M, Richardson DM 1996 What attributes make some plant species more invasive? Ecology 77, 1655–1661. (10.2307/2265768)

[74] Kueffer C, Pyšek P, Richardson DM 2013 Integrative invasion science: model systems, multi-site studies, focused meta-analysis, and invasion syndromes. New Phytol. 200, 615–633. (10.1111/nph.12415) 23879193

[75] Gabriele-Rivet V, Koffi JK, Pelcat Y, Arsenault J, Cheng A, Lindsay LR, Lysyk TJ, Rochon K, Ogden NH 2017 A risk model for the Lyme disease vector *Ixodes scapularis* (Acari: Ixodidae) in the Prairie Provinces of Canada. J. Med. Entomol. 54, 862–868. (10.1093/jme/tjx036) 28399276

[76] Ogden NH et al. 2006 Climate change and the potential for range expansion of the Lyme disease vector *Ixodes scapularis* in Canada. Int. J. Parasitol. 36, 63–70. (10.1016/j.ijpara.2005.08.016) 16229849

[77] Chiyaka C, Singer BH, Halbert SE, Morris JG Jr, van Bruggen AH. 2012 Modeling huanglongbing transmission within a citrus tree. Proc. Natl Acad. Sci. USA 24, 12 213–12 218. (10.1073/pnas.1208326109) PMC3409777 22783015

[78] Ogden NH et al. 2006 *Ixodes scapularis* ticks collected by passive surveillance in Canada: analysis of geographic distribution and infection with the Lyme borreliosis agent *Borrelia burgdorferi*. J. Med. Entomol. 43, 600–609. (10.1093/jmedent/43.3.600) 16739422

[79] Clow K, Leighton PA, Ogden NH, Lindsay LR, Michel P, Pearl D, Jardine C 2017 Northward range expansion of *Ixodes scapularis* evident over a short timescale in Ontario, Canada. PLoS ONE 12, e0189393 (10.1371/journal.pone.0189393) 29281675 PMC5744917

[80] Xie JY, Tang WJ, Yang YH 2018 Fish assemblage changes over half a century in the Yellow River, China. Ecol. Evol. 8, 4173–4182 (10.1002/ece3.3890) 29721289 PMC5916296

[81] Malek R, Tattoni C, Ciolli M, Corradini S, Andreis D, Ibrahim A, Mazzoni V, Eriksson A, Anfora G 2018 Coupling traditional monitoring and citizen science to disentangle the invasion of *Halyomorpha halys*. ISPRS Int. J. Geo-Inf. 7, 171 (10.3390/ijgi7050171)

[82] Brundu G, Richardson DM 2016 Planted forests and invasive alien trees in Europe: a code for managing existing and future plantings to mitigate the risk of negative impacts from invasions. Neobiota 30, 5–47. (10.3897/neobiota.30.7015)

[83] Estoup A, Guillemaud T 2010 Reconstructing routes of invasion using genetic data: why, how and so what? Mol. Ecol. 19, 4113–4130. (10.1111/j.1365-294X.2010.04773.x) 20723048

[84] Chin CS et al. 2011 The origin of the Haitian cholera outbreak strain. N. Engl. J. Med. 364, 33–42. (10.1056/NEJMoa1012928) 21142692 PMC3030187

[85] Underwood E, Ustin S, DiPietro D 2003 Mapping nonnative plants using hyperspectral imagery. Remote Sens. Environ. 86, 150–161. (10.1016/S0034-4257(03)00096-8)

[86] Kalluri S, Gilruth P, Rogers D, Szczur M 2007 Surveillance of arthropod vector-borne infectious diseases using remote sensing techniques: a review. PLoS Pathog. 3, e116 (10.1371/journal.ppat.0030116) PMC2042005 17967056

[87] Fine PE 1993 Herd immunity: history, theory, practice. Epidemiol. Rev. 15, 265–302. (10.1093/oxfordjournals.epirev.a036121) 8174658

[88] Wilson JRU, Richardson DM, Rouget M, Procheş S, Amis MA, Henderson L, Thuiller W 2007 Residence time and potential range: crucial considerations in modelling plant invasions. Divers. Distrib. 13, 11–22. (10.1111/j.1366-9516.2006.00302.x)

[89] Williamson M, Dehnen-Schmutz K, Kuhn I, Hill M, Klotz S, Milbau A, Stout J, Pyšek P 2009 The distribution of range sizes of native and alien plants in four European countries and the effects of residence time. Divers. Distrib. 15, 158–166. (10.1111/j.1472-4642.2008.00528.x)

[90] Gasso N, Pyšek P, Vila M, Williamson M 2010 Spreading to a limit: the time required for a neophyte to reach its maximum range. Divers. Distrib. 16, 310–311. (10.1111/j.1472-4642.2010.00647.x)

[91] Desprez-Loustau M-L, Robin C, Buée M, Courtecuisse R, Garbaye J, Suffert F, Sache I, Rizzo DM 2007 The fungal dimension of biological invasions. Trends Ecol. Evol. 22, 472–480. (10.1016/j.tree.2007.04.005) 17509727

[92] Cox R, Sanchez J, Revie CW 2013 Multi-criteria decision analysis tools for prioritising emerging or re-emerging infectious diseases associated with climate change in Canada. PLoS ONE 8, e68338 (10.1371/journal.pone.0068338) 23950868 PMC3737372

[93] van Kleunen M, Dawson W, Schlaepfer D, Jeschke JM, Fischer M. 2010 Are invaders different? A conceptual framework of comparative approaches for assessing determinants of invasiveness. Ecol. Lett. 13, 947–958. (10.1111/j.1461-0248.2009.01418.x) 20576028

[94] Cleaveland S, Laurenson MK, Taylor LH 2001 Diseases of humans and their domestic mammals: pathogen characteristics, host range, and the risk of emergence. Phil. Trans. R. Soc. B 356, 991–999. (10.1098/rstb.2001.0889) 11516377 PMC1088494

[95] Olival KJ, Hosseini PR, Zambrana-Torrelio C, Ross N, Bogich TL, Daszak P 2017 Host and viral traits predict zoonotic spillover from mammals. Nature 546, 646–650. (10.1038/nature22975) 28636590 PMC5570460

[96] Richardson DM, Pyšek P 2006 Plant invasions—merging the concepts of species invasiveness and community invasibility. Prog. Phys. Geogr. 30, 409–431. (10.1191/0309133306pp490pr)

[97] McKinney ML 2008 Effects of urbanization on species richness: a review of plants and animals. Urb. Ecosyst. 11, 161–176. (10.1007/s11252-007-0045-4)

[98] Winemiller KO, Fitzgerald DB, Bower LM, Pianka ER 2015 Functional traits, convergent evolution, and periodic tables of niches. Ecol. Lett. 18, 737–751. (10.1111/ele.12462) 26096695 PMC4744997

[99] Rouget M, Robertson MP, Wilson JRU, Hui C, Essl F, Renteria JL, Richardson DM 2016 Invasion debt—quantifying future biological invasions. Divers. Distrib. 22, 445–456. (10.1111/ddi.12408)

[100] Burgman MA, McCarthy MA, Robinson A, Hester SM, McBride MF, Elith J, Panetta FD 2013 Improving decisions for invasive species management: reformulation and extensions of the Panetta-Lawes eradication graph. Divers. Distrib. 19, 603–607. (10.1111/ddi.12055)

[101] Wilson JR, Panetta FD, Lindgren C 2017 Detecting and responding to alien plant incursions. Cambridge, UK: Cambridge University Press.

[102] Ogden NH, Koffi JK, Pelcat Y, Lindsay LR 2014 Environmental risk from Lyme disease in central and eastern Canada: a summary of recent surveillance information. Can. Commun. Dis. Rep. 40, 74–82. (10.14745/ccdr.v40i05a01) 29769885 PMC5864485

[103] Ruder MG, Lysyk TJ, Stallknecht DE, Foil LD, Johnson DJ, Chase CC, Dargatz DA, Gibbs EP 2015 Transmission and epidemiology of bluetongue and epizootic hemorrhagic disease in North America: current perspectives, research gaps, and future directions. Vector Borne Zoonotic Dis. 15, 348–363. (10.1089/vbz.2014.1703) 26086556

[104] Gilmour MW, Graham M, Reimer A, Van Domselaar G. 2013 Public health genomics and the new molecular epidemiology of bacterial pathogens. Publ. Health Genom. 16, 25–30. (10.1159/000342709) 23548714

[105] Ficetola GF, Gentile F, Bonin A, Miaud C 2008 Population genetics reveals origin and number of founders in a biological invasion. Mol. Ecol. 17, 773–782. (10.1111/j.1365-294X.2007.03622.x) 18194168

[106] Berthouly-Salazar C, Hui C, Blackburn TM, Gaboriaud C, Van Rensburg BJ, Van Vuuren BJ, Le Roux JJ. 2013 Long-distance dispersal maximizes evolutionary potential during rapid geographic range expansion. Mol. Ecol. 22, 5793–5804. (10.1111/mec.12538) 24192018

[107] Diedericks G, Henriques R, von der Heyden S, Weyl OLF, Hui C 2018 The ghost of introduction past: spatial and temporal variability in the genetic diversity of invasive smallmouth bass. Evol. Appl. 11, 1609–1629. (10.1111/eva.12652) 30344631 PMC6183467

[108] Chown SL, Hodgins KA, Griffin PC, Oakeshott JG, Byrne M, Hoffmann AA 2015 Biological invasions, climate change and genomics. Evol. Appl. 8, 23–46. (10.1111/eva.12234) 25667601 PMC4310580

[109] Jerde CL, Mahon AR, Chadderton WL, Lodge DM 2011 ‘Sight-unseen’ detection of rare aquatic species using environmental DNA. Conserv. Lett. 4, 150–157. (10.1111/j.1755-263X.2010.00158.x)

[110] Goolsby JA, DE Barro PJ, Makinson JR, Pemberton RW, Hartley DM, Frohlich DR. 2006 Matching the origin of an invasive weed for selection of a herbivore haplotype for a biological control programme. Mol. Ecol. 15, 287–297. (10.1111/j.1365-294X.2005.02788.x) 16367847

[111] Ogden NH, Abdelmalik P, Pulliam JRC 2017 Emerging infectious diseases: prediction and detection. Can. Commun. Dis. Rep. 43, 206–211. (10.14745/ccdr.v43i10a03) 29770047 PMC5764723

[112] Watson M, Shaw D, Molchanoff L, McInnes C 2009 Challenges, lessons learned and results following the implementation of a human papilloma virus school vaccination program in South Australia. Aust. N. Z. J. Public Health 33, 365–370. (10.1111/j.1753-6405.2009.00409.x) 19689598

[113] Hulme PE, Pyšek P, Nentwig W, Measures P 2009 Will threat of biological invasions unite the European Union? Science 324, 40–41. (10.1126/science.1171111) 19342572

[114] Schmitz DC, Simberloff D 2001 Needed: a national center for biological invasions. Issues Sci. Technol. 17, 57–62.

[115] Latombe G et al. 2017 A vision for global monitoring of biological invasions. Biol. Conserv. 213, 295–308. (10.1016/j.biocon.2016.06.013)

[116] Dion M, AbdelMalik P, Mawudeku A 2015 Big data and the Global Public Health Intelligence Network (GPHIN). Can. Commun. Dis. Rep. 41, 209–214. (10.14745/ccdr.v41i09a02) 29769954 PMC5933838

[117] Drawert B, Griesemer M, Petzold LR, Briggs CJ 2017 Using stochastic epidemiological models to evaluate conservation strategies for endangered amphibians. J. R. Soc. Interface 14, 20170480 (10.1098/rsif.2017.0480) 28855388 PMC5582134

[118] Muirhead J, Leung B, Van Overdijk C, Kelly D, Nandakumar K, Marchant K, MacIsaac H 2006 Modelling local and long-distance dispersal of invasive emerald ash borer *Agrilus planipennis* (Coleopteran) in North America. Divers. Distrib. 12, 71–79. (10.1111/j.1366-9516.2006.00218.x)

[119] Wonham MJ, de-Camino-Beck T, Lewis MA 2004 An epidemiological model for West Nile virus: invasion analysis and control applications. Proc. R. Soc. B 271, 501–507. (10.1098/rspb.2003.2608) PMC1691622 15129960

[120] Reisen WK 2013 Ecology of West Nile virus in North America. Viruses 5, 2079–2105. (10.3390/v5092079) 24008376 PMC3798891

[121] Packer JG et al. 2017 Global network for invasion science: benefits, challenges and guidelines. Biol. Invasions 19, 1081–1096. (10.1007/s10530-016-1302-3)

